# Longitudinally Extensive Transverse Myelitis as a First Manifestation of Sarcoidosis

**DOI:** 10.7759/cureus.44037

**Published:** 2023-08-24

**Authors:** Ricardo A Rodrigues, Telma Alves, João A Sousa, Andre Jorge, Argemiro Geraldo

**Affiliations:** 1 Internal Medicine, University Hospital Center of Coimbra, Coimbra, PRT; 2 Neurology, University Hospital Center of Coimbra, Coimbra, PRT

**Keywords:** granulomatous disease, thoracic lymphadenopathies, spinal cord sarcoidosis, spinal cord diseases, neurologic sarcoidosis, longitudinally extensive transverse myelitis, sarcoidosis

## Abstract

Longitudinally extensive transverse myelitis (LETM) is a debilitating inflammatory spinal cord lesion involving several spinal segments. There are several possible etiologies, with spinal cord sarcoidosis being a rare cause of LETM. Spinal cord sarcoidosis is, in itself, a rare manifestation of sarcoidosis that can be difficult to diagnose, especially in patients with no prior history of systemic sarcoidosis, frequently leading to a delayed diagnosis. We report the case of a 53-year-old man who developed LETM as the first manifestation of sarcoidosis. The patient presented with progressive lower limb weakness, urinary retention, sensory disturbances, and muscle spasms. Imaging studies showed hyperintense lesions extending over multiple spinal segments. After the exclusion of other causes and a lymph node biopsy showing non-caseating granulomas, the diagnosis of LETM secondary to sarcoidosis was confirmed.

## Introduction

Longitudinally extensive transverse myelitis (LETM) is an inflammatory spinal cord lesion extending over three or more vertebral segments. Clinical presentation is varied depending on the affected area and can include gait, sexual, gastrointestinal, or urinary sphincter dysfunction, sensory disturbances, and paraparesis or tetraparesis. The onset of symptoms is often sudden, and most patients have a poor prognosis that can range from permanent disabilities to death from respiratory failure [1]. LETM has been linked to a variety of causes, including inflammatory disorders, cancer, infections, and metabolic diseases [2]. Sarcoidosis is a rare cause of LETM, with less than 1% of sarcoidosis patients developing spinal cord involvement, with even less as the first manifestation [3]. We report the case of a 53-year-old male patient presenting with extensive longitudinal transverse myelitis as the first manifestation of sarcoidosis.

## Case presentation

**Figure 1:**
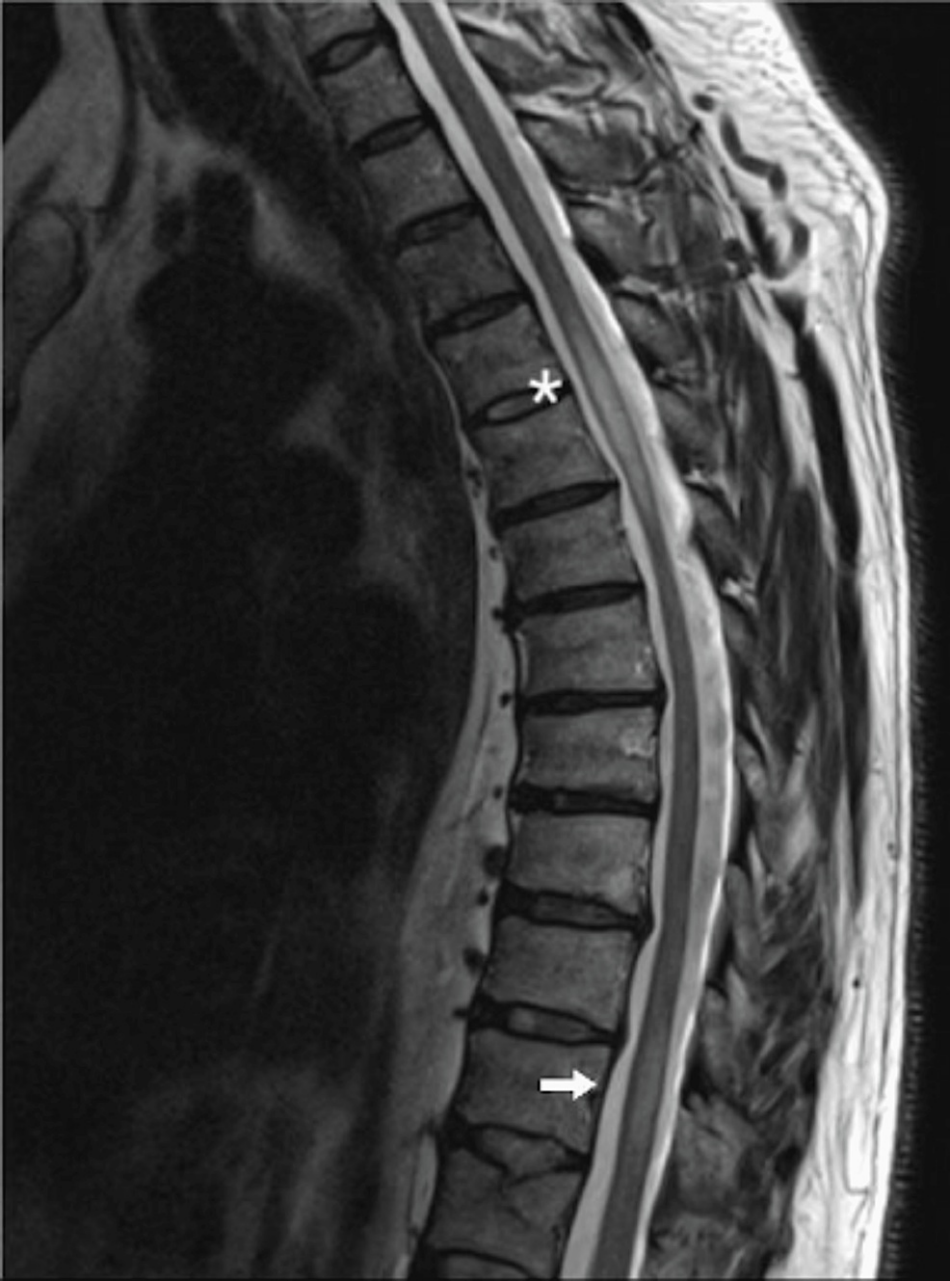
Spinal MRI imaging Spinal MRI T2-weighted image showing hyperintense lesion extending from D4 to D6 (asterisk) and in the transition of D10-D11 (arrow)

A 53-year-old man with no prior medical history was admitted to the emergency department with complaints of bilateral lower limb weakness. The first symptoms began ten months prior with complaints of urinary retention, followed by mild lower limb muscular weakness two months later. The patient showed progressive worsening of symptoms until losing the ability to walk. Additionally, the patient reported lower back pain, decreased lower limb sensitivity, and muscle spasms in the right leg. Neurological examination showed a relatively symmetric paraparesis with grade 3 strength on hip flexion, 3 on leg extension, 2 on flexion, and 1 on foot dorsiflexion, with hyperreflexia at patellar and Achilles myotatic reflexes. Magnetic resonance imaging (MRI) confirmed the diagnosis of longitudinally extensive transverse myelitis extending from D4 to D6 and D10 to D12 with no other significant changes (Figure 1).

The patient was admitted to the neurology ward and was started on high-dose corticosteroids (1000 mg of methylprednisolone) while the etiological study continued. Lumbar puncture showed a slight increase in leukocytes (16/mm3, mainly mononuclear) and proteins (64 mg/dL), with no other changes. IgM antibodies for Borrelia were present in the patients' blood work. Although the patient had no suggestive epidemiological history or signs and symptoms of Lyme disease, doxycycline was started. After consulting infectious diseases specialists, the antibiotic regimen was changed to ceftriaxone for better central nervous system (CNS) coverage, while confirmatory phase 1 and phase 2 antibodies and Borrelia burgdorferi DNA detection in cerebrospinal fluid (CSF) using polymerase chain reaction (PCR) were assessed. There were no other changes in the patient's blood work, including infectious serology and cultures, an autoimmune study (with negative aquaporin-4 and myelin oligodendrocyte glycoprotein [MOG] antibodies), or protein electrophoresis.

After five days of high-dose corticosteroids, only a negligible improvement in lower limb weakness was observed, and plasmapheresis was started. At the end of seven every other day plasma exchanges, there was only a mild improvement in leg extension (grade 3 strength to 4+) with no change in the remaining neurological exams. Confirmatory tests for Lyme disease were not suggestive of an active or recent infection. The first results were assumed to be a possible cross-reaction, and antibiotics were stopped.

**Figure 2:**
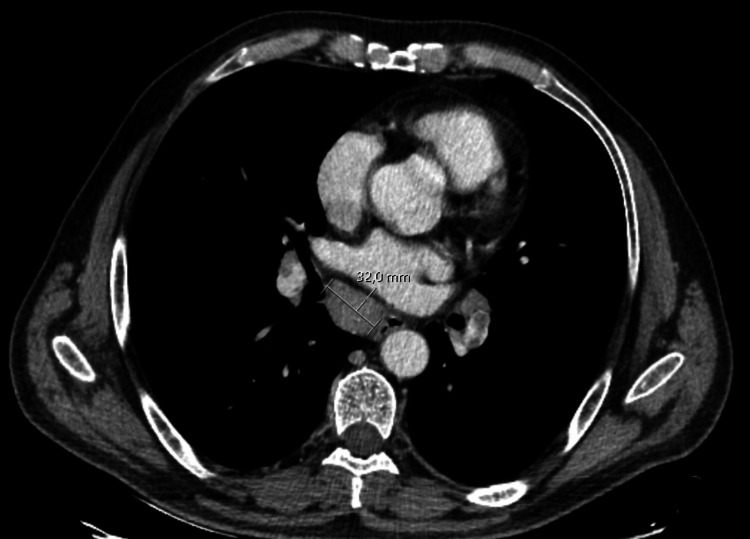
Thoracic CT imaging Computer tomography imaging of the thorax, showing lymphadenopathies at the base of the trachea measuring 32 mm × 23 mm

It was decided to start five days of intravenous immunoglobulin therapy while complemental investigation was continued with a full-body computer tomography (CT) to exclude occult malignancy and possible paraneoplastic syndrome. A CT scan showed multiple thoracic lymphadenopathies at the base of the trachea and in both lung hilums, the biggest measuring 32 mm × 23 mm, with no other significant changes (Figure 2).

A lymph node biopsy was performed using mediastinoscopy by thoracic surgery, with histological studies showing non-necrotizing granulomas with giant Langhan cells, giving us the diagnosis of sarcoidosis. The case was discussed with the autoimmune diseases team, and the patient was started on prednisolone 1 mg/kg/day for one month, followed by a long taper (5 mg reduction every two weeks) and methotrexate 15 mg/week.

The patient was later transferred to a rehabilitation clinic, maintaining neurology and autoimmune outpatient follow-up. At the time of hospital discharge, the clinical picture remained stable, with a slight improvement in leg and foot flexion. One year later, the patient maintained lower limb weakness with grade 2 on flexion and grade 3/4 on extension. He is currently being treated with methotrexate (15 mg weakly) and infliximab, which was later started due to a lack of response to previous medication. So far, the patient has not shown any other clinical manifestations of sarcoidosis.

## Discussion

Sarcoidosis is a multisystem granulomatous disease with non-caseating granulomas present in the involved organs. CNS involvement is rare, affecting approximately 5% to 15% of sarcoidosis patients, with spinal cord involvement affecting less than 1% [3,4]. Neurosarcoidosis can affect multiple sites; the most frequent manifestations are cranial nerve involvement, especially optic neuritis and facial nerve palsy. Other possible presentations include aseptic meningitis, peripheral neuropathy, and, less commonly, pituitary and spinal cord dysfunction [3,5]. We report the case of longitudinally extensive transverse myelitis in a patient with no previous diagnosis of sarcoidosis.

The gold standard for the diagnosis of neurosarcoidosis is a CNS biopsy showing non-caseating granulomas, with no other less invasive method having such good sensitivity or specificity [3]. This usually leads to a delay in diagnosis, but only after the exclusion of other possible cases. The differential diagnosis of spinal cord sarcoidosis depends on the clinical presentation, imaging features, and biopsy results. Some of the possible conditions that can mimic spinal cord sarcoidosis are multiple sclerosis, neuromyelitis optica, spinal cord tumors, spinal arteriovenous fistulae, paraneoplastic syndromes, tuberculous leptomeningitis, and Erdheim-Chester disease [6].

Cerebrospinal fluid analysis usually shows increased protein levels and cell count, with some cases reporting hypoglycorrhachia [7]. CSF angiotensin-converting enzyme (ACE) levels are not sensitive but may be specific [8]. Hyperintensity on T2-weighted images with gadolinium enhancement is often seen in MRI, and the unique and characteristic ‘trident sign’ on medullary axial views, although specific, is frequently absent [9,10].

These nonspecific findings and the need to exclude other possible causes make the diagnosis of spinal cord sarcoidosis challenging, particularly in patients with no previous history of systemic sarcoidosis, where the final diagnosis is often delayed. In our case, despite not having performed a CNS biopsy, we could only assume the diagnosis of neurosarcoidosis after excluding other possible causes and making the new diagnosis of sarcoidosis due to a lymph node biopsy and suggestive changes in CSF analysis and MRI. The case was further complicated by the initial result suggesting Lyme disease and possible neuroborreliosis, with the exclusion of this diagnosis only being made after the negative confirmatory tests.

Treatment is based on glucocorticoids as the first-line agents, with the dose and duration depending on the severity and response to therapy. Other immunomodulatory therapies, such as methotrexate, mycophenolate mofetil, and cyclophosphamide, should be used in patients with contraindications to glucocorticoid treatment [5,11]. They can be considered early in the course of the disease in patients with high-dose glucocorticoid treatment and with manifestations that will likely require a long course of glucocorticoids. Infliximab is an antihuman antibody that has been associated with symptom relief and regression of neurologic deficits in glucocorticoid-refractory neurosarcoidosis [12]. The prognosis of neurosarcoidosis depends on the severity and location of inflammation, as well as the response to therapy [3,5].

## Conclusions

This case illustrates the importance of a thorough etiological investigation in patients with LETM, which is a serious and potentially fatal condition. Spinal cord sarcoidosis is a rare but possible cause of LETM that requires histological confirmation of sarcoidosis with non-caseating granulomas in the CNS or other organs. Adjuvant diagnostic methods such as CSF analysis, MRI, and ACE levels are neither specific nor sensitive but may help to rule out other causes. Therefore, clinicians should maintain a high index of suspicion for spinal cord sarcoidosis in patients with LETM; spinal cord sarcoidosis should not be ruled out as a potential cause even in patients without prior sarcoidosis diagnosis.
